# Supplementary material for: Lifelong Machine Learning Potentials
Source: J Chem Theory Comput. 2023 Jun 8;19(12):3509–25. doi: 10.1021/acs.jctc.3c00279 (PMC10308836; doi:10.1021/acs.jctc.3c00279)
Supplement: Supplementary file 1 — ct3c00279_si_001.pdf [file ct3c00279_si_001.pdf]

Supporting Information: Lifelong Machine Learning Potentials

Marco Eckhoff* and Markus Reiher†
ETH Zürich, Departement Chemie und Angewandte Biowissenschaften, Vladimir-Prelog-Weg 2, 8093 Zürich, Switzerland.
(Dated: May 11, 2023)

S1. SUPPORTING COMPUTATIONAL DETAILS

S1.1. Activation Function and Weight Initialization

For the input and hidden layers of the atomic neural networks, we propose the activation function

$$f(x) = 1.59223 \cdot \tanh(x)\ . \quad (1)$$

This activation function will target to yield a standard deviation of unity for the neuron values in each layer before and after the application of the activation function if the input values of the atomic neural network are normally distributed.

For this purpose, the weights a need to be initialized as

$$a_{\kappa\lambda}^{m,\mu\nu} = \begin{cases} \text{random}\left(\pm\dfrac{\sqrt{3}}{\sqrt{n_\mu}}\right) & \text{for } \nu \neq \nu_{\max} \\ \text{random}\left(\pm\dfrac{\text{std}\left(\frac{\mathbf{E}^{\text{train}}}{\mathbf{N}_{\text{atom}}^{\text{train}}}\right)}{0.885\sqrt{n_\mu}}\right) & \text{otherwise} \end{cases} . \quad (2)$$

The random function returns a uniformly distributed random number in the given interval. n_μ is the number of neurons in the first of the two connected layers μ, which equals the number of input values for each neuron in the second layer ν. The weights connecting the last hidden layer and the output neuron, i.e., those with index $\nu_{\max}$, can be adjusted to match the standard deviation of the training energies per atom. The range of the random numbers is divided by 0.885 because this value is the mean neuron value after application of the activation function for normally distributed input.

For $\nu \neq \nu_{\max}$ the biases $b_\lambda^{m,\nu}$ can be initialized as zero. To align the mean energy of the training data and that of the untrained atomic neural networks, the bias weight of each output layer $\nu_{\max}$ needs to be initialized as mean energy per atom of the respective element m [1, 2]. This energy can be obtained from a least-squares fit of the energies per atom as a function of the stoichiometries. If the relative stoichiometries of some elements are not varied in the training data set, these elements can be grouped in the calculation of the energies per atom.

In this work, the weight initialization of α_i^m and β_i^m was restricted to values inside the interval $[-10, 10]$.

S1.2. Free Atom Energies

Table S1: Atomic DFT energies E^{ref} in Hartree (E_h) of the neutral free atoms in their lowest spin state.

Atom	$E^{\text{ref}}\ /\ E_\text{h}$
H	−0.49961798
C	−37.79598522
N	−54.53215363
O	−75.00969423
F	−99.66915592
S	−397.93838803
Cl	−459.95846673
Se	−2401.12499845
Br	−2573.72084670
I	−297.66553177

S1.3. Atom-Centered Symmetry Function Parameters

Table S2: All combinations of the listed parameters were applied for angular ACSFs. All radial and angular ACSFs were constructed for every element pair and triple, respectively. The cutoff radius was set to $R_\text{c} = 12$ Å for all ACSFs.

Radial ACSFs	
$\eta^{\text{rad}}\ /\ \text{Å}^{-2}$	0, 0.006057, 0.014224, 0.027869, 0.041917, 0.065572, 0.110664, 0.214650, 0.554219
**Angular ACSFs**	
$\eta^{\text{ang}}\ /\ \text{Å}^{-2}$	0.002368, 0.055330
λ	−1, 1
ξ	1, 2.409421, 9.996864

* marco.eckhoff@phys.chem.ethz.ch
† mreiher@ethz.ch

S2. SUPPORTING RESULTS

S2.1. Performance Evaluation of Activation Function and Weight Initialization

Table S3: RMSE values of individual HDNNPs and the ensemble trained on reference data set B using the activation function $\tanh(x)$ and uniformly distributed random initial weights $a_{\kappa\lambda}^{\mu\nu}$ and b_{λ}^{ν} from the interval $[-1/\sqrt{n_\mu}, 1/\sqrt{n_\mu})$. The data are compared with results using the activation function $1.59223 \cdot \tanh(x)$ and the tailored weight initialization described in Section S1.1, which were also employed in all other trainings. The CoRe optimizer and lifelong adaptive data selection were applied for 2000 epochs.

Individual HDNNPs	$\tanh(x)$	$1.59223 \cdot \tanh(x)$, tailored weight init.
$\mathrm{RMSE}(E^{\mathrm{train}})$ / meV atom^{-1}	5.9 ± 0.4	3.9 ± 0.4
$\mathrm{RMSE}(E^{\mathrm{test}})$ / meV atom^{-1}	6.4 ± 0.4	4.5 ± 0.6
$\mathrm{RMSE}(F_{\alpha,n}^{\mathrm{train}})$ / meV Å^{-1}	134 ± 7	99 ± 7
$\mathrm{RMSE}(F_{\alpha,n}^{\mathrm{test}})$ / meV Å^{-1}	145 ± 7	116 ± 4
Ensemble		
$\mathrm{RMSE}(\overline{E})$ / meV atom^{-1}	3.7	2.6
$\mathrm{RMSE}(\overline{F}_{\alpha,n})$ / meV Å^{-1}	87	64

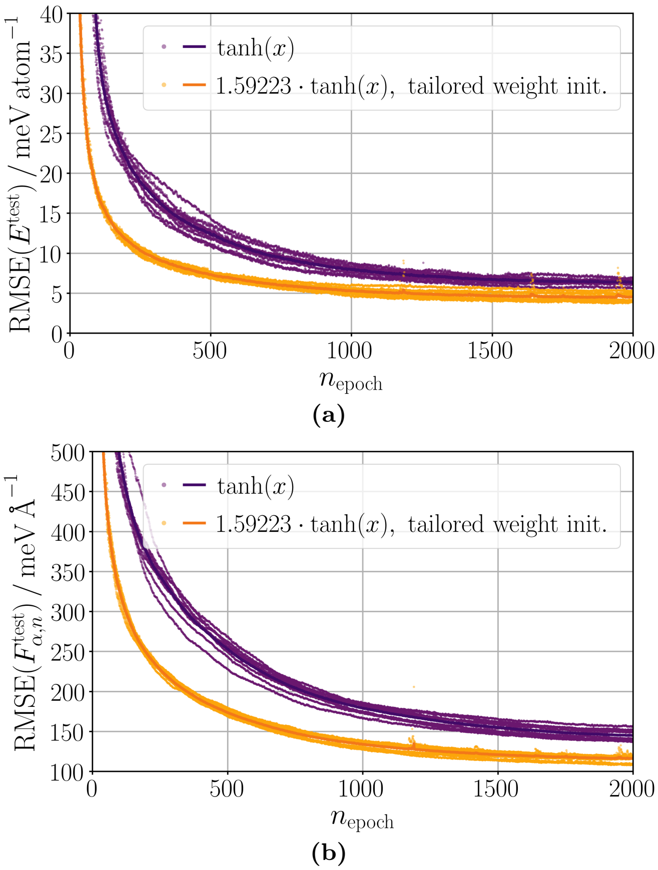

Figure S1: Convergence for training reference data set B using the activation function $\tanh(x)$ and uniformly distributed random initial weights $a_{\kappa\lambda}^{\mu\nu}$ and b_{λ}^{ν} from the interval $[-1/\sqrt{n_\mu}, 1/\sqrt{n_\mu})$. The data are compared with results using the activation function $1.59223 \cdot \tanh(x)$ and the tailored weight initialization described in Section S1.1, which were also employed in all other trainings. The test set RMSE values of **(a)** energies E^{test} and **(b)** atomic force components $F_{\alpha,n}^{\mathrm{test}}$ are shown as a function of the training epoch n_{epoch}. RMSE values of individual HDNNPs are represented by dots, while their mean is shown by a solid line. The CoRe optimizer and lifelong adaptive data selection were applied.

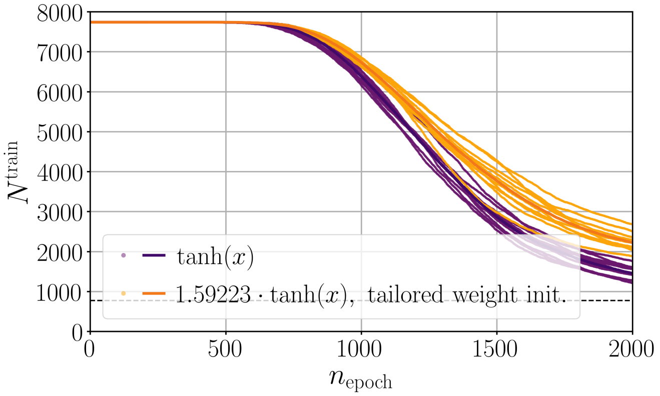

Figure S2: Training data set reduction using the CoRe optimizer and different activation functions and weight initializations (see caption of Figure S1) for training reference data set B. The number of considered training conformations N^{train} is shown as a function of the training epoch n_{epoch}. The values of N^{train} of individual HDNNPs are represented by dots, while their mean is shown by a solid line. The black dashed line represents the number of training conformations which was used for fitting in each epoch.

S2.2. Energy Distribution of Reference Data

Table S4: The mean energy ranges $\overline{E}^{\text{ref,methyl}}_{\text{S}_\text{N}\text{2,range}}$ and $\overline{E}^{\text{ref,butyl}}_{\text{S}_\text{N}\text{2,range}}$ and mean standard deviations $\overline{E}^{\text{ref,methyl}}_{\text{S}_\text{N}\text{2,std}}$ and $\overline{E}^{\text{ref,butyl}}_{\text{S}_\text{N}\text{2,std}}$ for the individual $\text{S}_\text{N}2$ reaction systems containing a central methyl or *tert*-butyl carbon atom in the reference data sets A, B, and C.

Reference data set	A	B	C
$\overline{E}^{\text{ref,methyl}}_{\text{S}_\text{N}\text{2,range}}$ / meV atom^{-1}	805.3	746.6	911.9
$\overline{E}^{\text{ref,methyl}}_{\text{S}_\text{N}\text{2,std}}$ / meV atom^{-1}	171.6	164.5	172.1
$\overline{E}^{\text{ref,butyl}}_{\text{S}_\text{N}\text{2,range}}$ / meV atom^{-1}	460.4	437.8	437.8
$\overline{E}^{\text{ref,butyl}}_{\text{S}_\text{N}\text{2,std}}$ / meV atom^{-1}	96.3	89.6	89.6

S2.3. Prediction Error Distribution for Reference Data Set A

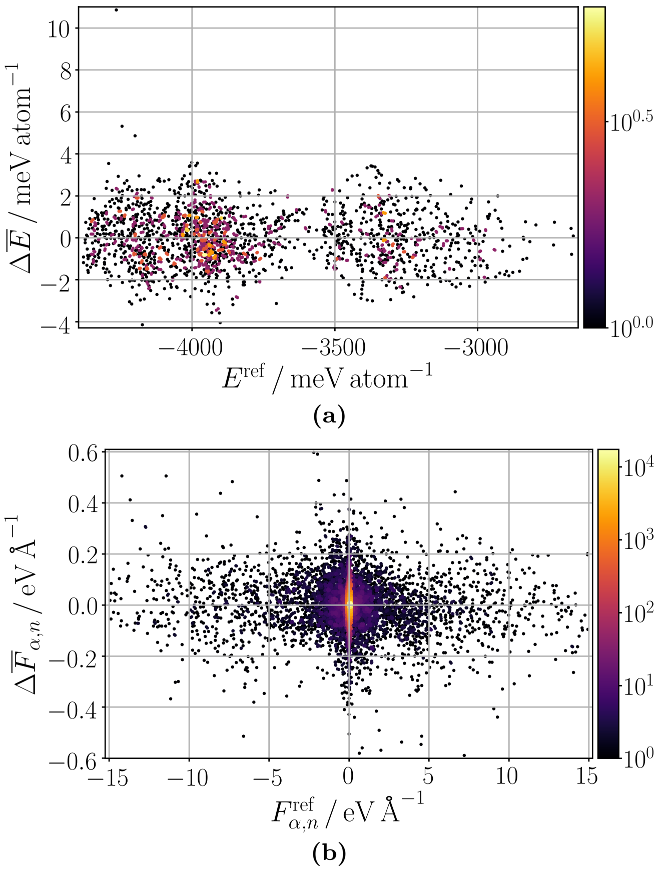

Figure S3: Errors with respect to the DFT reference of reference data set A for the ensemble prediction of **(a)** energies $\Delta\overline{E}$ and **(b)** atomic force components $\Delta\overline{F}_{\alpha,n}$. The errors are shown as a function of the respective DFT reference E^{ref} and $F^{\text{ref}}_{\alpha,n}$. The color represents the data point density in a grid of size 200×133, which covers the plot area. The training was performed with the CoRe optimizer and lifelong adaptive data selection for 2000 epochs using eeACSFs.

S2.4. Training Process for Different Structural Representations

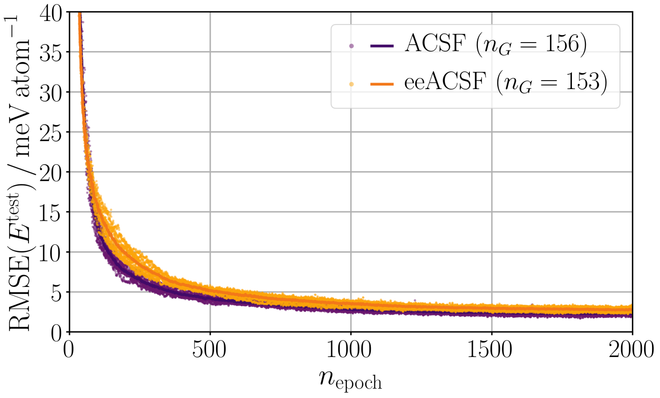

(a)

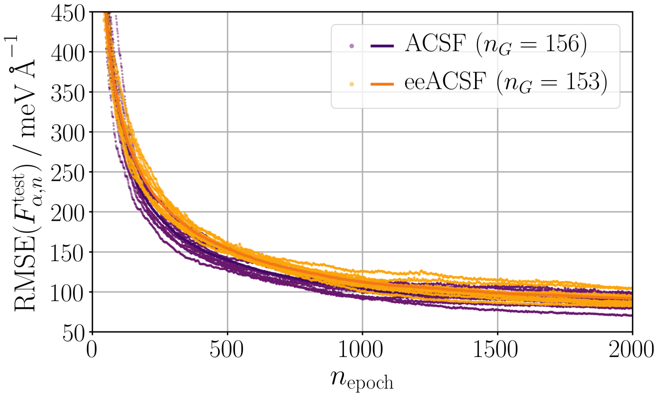

(b)

Figure S4: Convergence for training reference data set A with ACSF vectors of size $n_G = 156$ and eeACSF vectors of size $n_G = 153$. The test set RMSE values of **(a)** energies E^{test} and **(b)** atomic force components $F^{\text{test}}_{\alpha,n}$ are shown as a function of the training epoch n_{epoch}. RMSE values of individual HDNNPs are represented by dots, while their mean is shown by a solid line. The CoRe optimizer and lifelong adaptive data selection were applied.

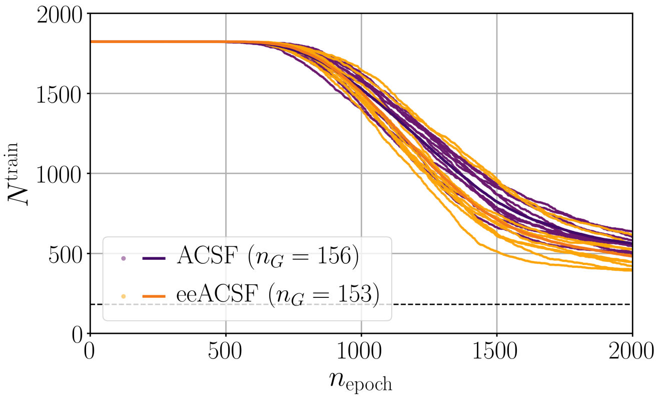

Figure S5: Training data set reduction using the CoRe optimizer and ACSFs or eeACSFs for training reference data set A. The number of considered training conformations N^{train} is shown as a function of the training epoch n_{epoch}. The values of N^{train} of individual HDNNPs are represented by dots, while their mean is shown by a solid line. The black dashed line represents the number of training conformations which was used for fitting in each epoch.

S2.5. Prediction Error Distribution for Reference Data Set B

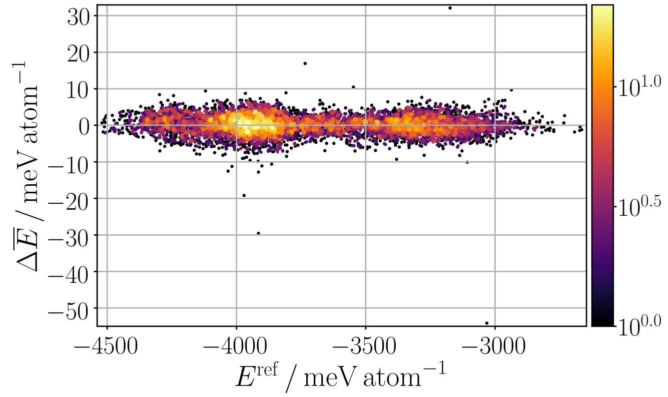

(a)

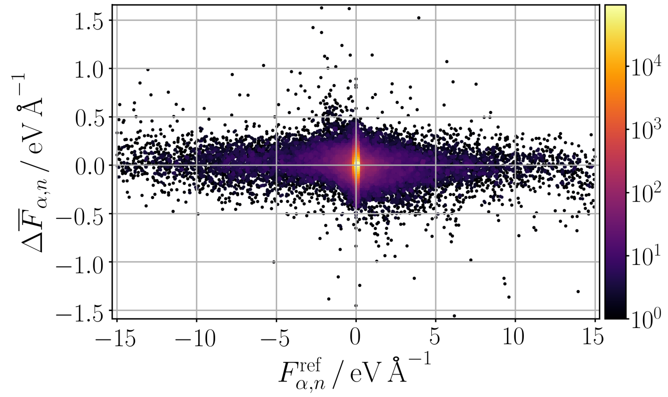

(b)

Figure S6: Errors with respect to the DFT reference of reference data set B for the ensemble prediction of **(a)** energies $\Delta\overline{E}$ and **(b)** atomic force components $\Delta\overline{F}_{\alpha,n}$. The errors are shown as a function of the respective DFT reference E^{ref} and $F^{\mathrm{ref}}_{\alpha,n}$. The color represents the data point density in a grid of size 200×133, which covers the plot area. The training was performed with the CoRe optimizer and lifelong adaptive data selection for 2000 epochs.

S2.6. Performance Evaluation of Random Data Selection

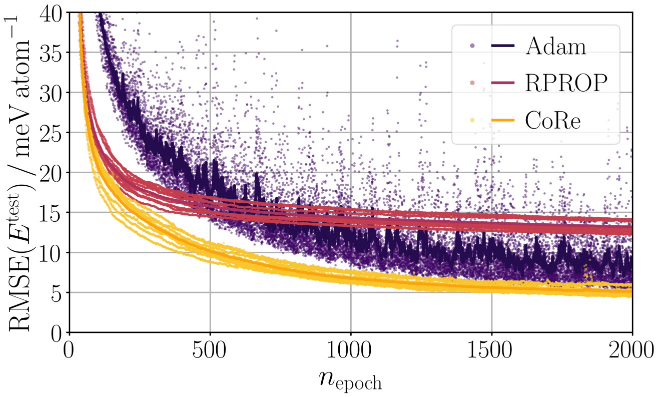

(a)

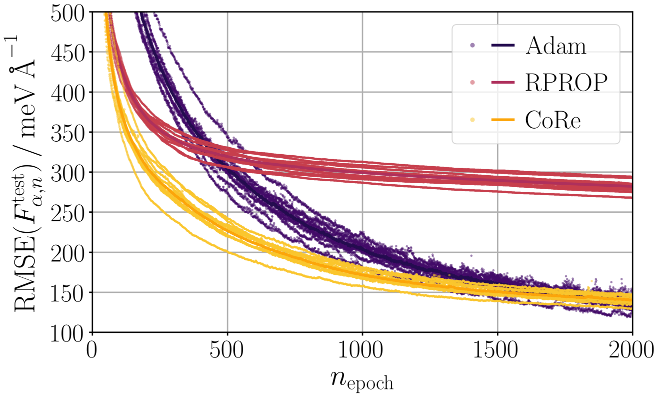

(b)

Figure S7: Convergence of the optimizers Adam, RPROP, and CoRe with random data selection for training reference data set B. The test set RMSE values of **(a)** energies E^{test} and **(b)** atomic force components $F^{\mathrm{test}}_{\alpha,n}$ are shown as a function of the training epoch n_{epoch}. RMSE values of individual HDNNPs are represented by dots, while their mean is shown by a solid line.

Table S5: RMSE values of individual HDNNPs and the ensemble trained on reference data set B using the optimizers RPROP, Adam, and CoRe. The optimizers and random data selection were applied for 2000 epochs.

Individual HDNNPs	RPROP	Adam	CoRe
RMSE(E^{train}) / meV atom^{-1}	12.9 ± 0.8	8.2 ± 2.1	4.3 ± 0.2
RMSE(E^{test}) / meV atom^{-1}	13.2 ± 0.8	8.5 ± 2.1	5.1 ± 0.5
RMSE($F_{\alpha,n}^{\mathrm{train}}$) / meV Å^{-1}	273 ± 9	127 ± 6	122 ± 4
RMSE($F_{\alpha,n}^{\mathrm{test}}$) / meV Å^{-1}	282 ± 8	137 ± 7	140 ± 6
Ensemble			
RMSE($\overline{E}$) / meV atom^{-1}	11.4	4.3	3.4
RMSE($\overline{F}_{\alpha,n}$) / meV Å^{-1}	236	106	95

S2.7. Training Process for Lifelong Learning Reference Data Set B

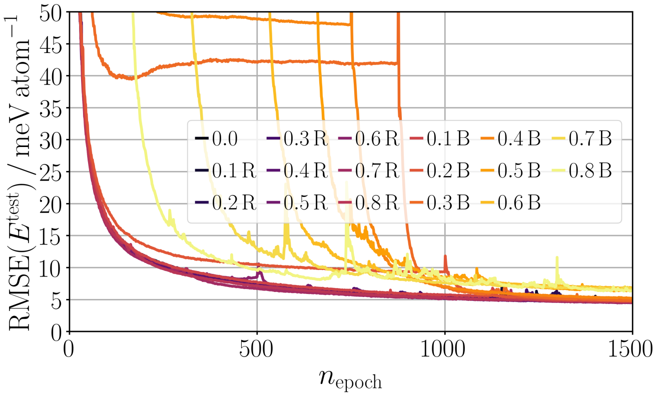

(a)

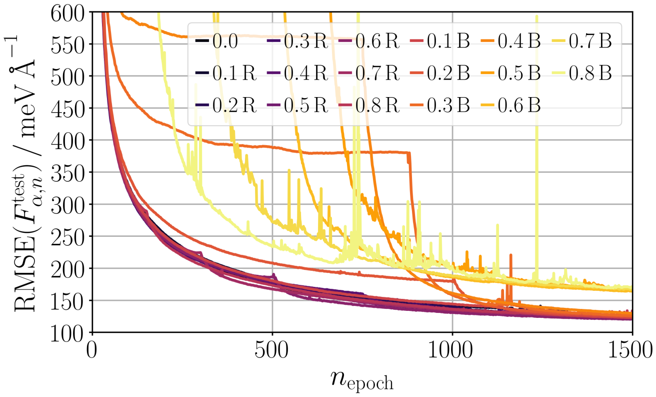

(b)

Figure S8: Convergence for training reference data set B applying lifelong learning with different late data fractions p_{late} and selection schemes ("Random" or "Block"). The test set RMSE values of **(a)** energies E^{test} and **(b)** atomic force components $F_{\alpha,n}^{\text{test}}$ are shown as a function of the training epoch n_{epoch}. The test set contains 10% of the conformations of the full reference data set B in every epoch, which were randomly chosen. Due to lifelong learning some sections of the test conformation space can be first trained at a late epoch explaining the step-like convergence graphs. RMSE values of individual HDNNPs are represented by dots, while their mean is shown by a solid line. The CoRe optimizer and lifelong adaptive data selection were applied.

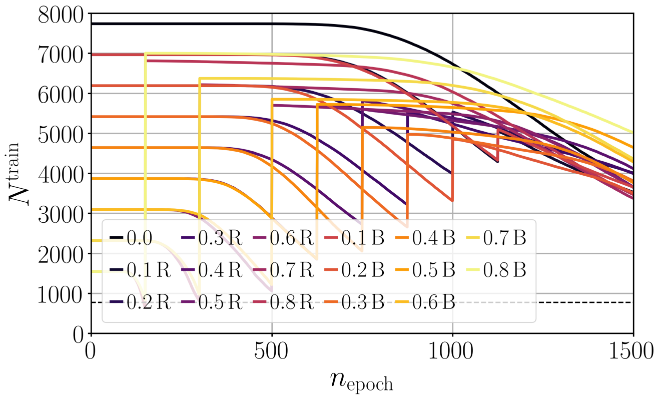

Figure S9: Training data set reduction using the CoRe optimzer and lifelong learning with different late data fractions p_{late} and selection schemes ("Random" or "Block") for training reference data set B. The number of considered training conformations N^{train} is shown as a function of the training epoch n_{epoch}. The values of N^{train} of individual HDNNPs are represented by dots, while their mean is shown by a solid line. The black dashed line represents the number of training conformations which was used for fitting in each epoch.

S2.8. Training Process for Lifelong Learning Reference Data Set C

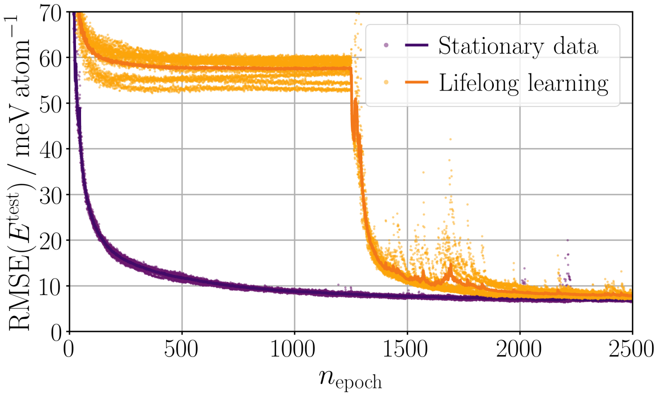

(a)

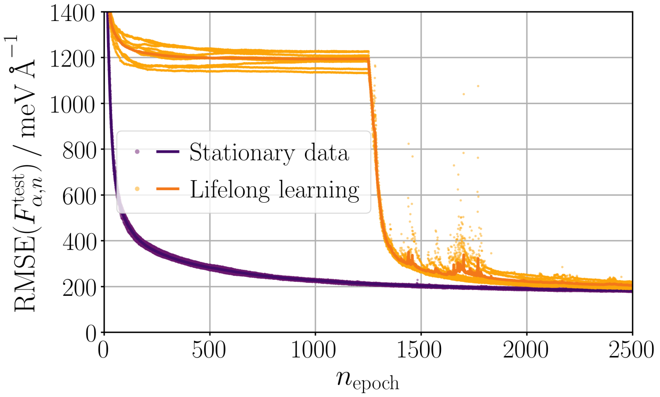

(b)

Figure S10: Convergence for training reference data set C with learning on a stationary batch of all training data and lifelong learning. The test set RMSE values of **(a)** energies E^{test} and **(b)** atomic force components $F_{\alpha,n}^{\text{test}}$ are shown as a function of the training epoch n_{epoch}. The test set contains 10% of the conformations of the full reference data set C in every epoch, which were randomly chosen. Due to lifelong learning some sections of the test conformation space can be first trained at a late epoch explaining the step-like convergence graphs. RMSE values of individual HDNNPs are represented by dots, while their mean is shown by a solid line. The CoRe optimizer and lifelong adaptive data selection were applied.

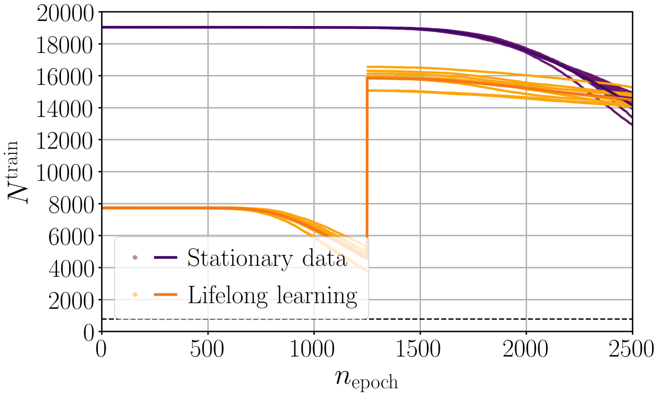

Figure S11: Training data set reduction using the CoRe optimizer and learning on a stationary batch of all training data or lifelong learning for training reference data set C. The number of considered training conformations N^{train} is shown as a function of the training epoch n_{epoch}. The values of N^{train} of individual HDNNPs are represented by dots, while their mean is shown by a solid line. The black dashed line represents the number of training conformations which was used for fitting in each epoch.

S2.9. Prediction Error Distribution for Reference Data Set C

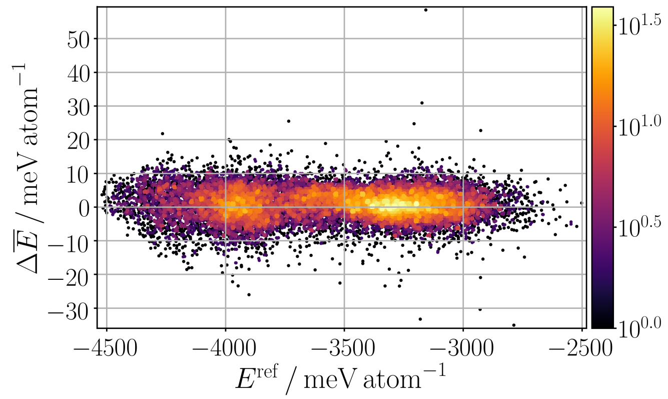

(a)

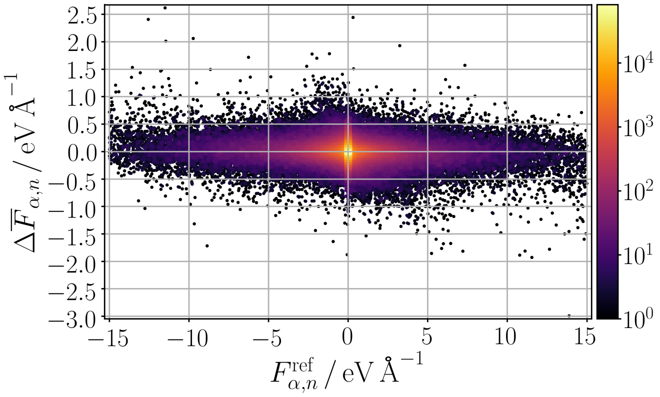

(b)

Figure S12: Errors with respect to the DFT reference of reference data set C for the ensemble prediction of **(a)** energies $\Delta\overline{E}$ and **(b)** atomic force components $\Delta\overline{F}_{\alpha,n}$. The errors are shown as a function of the respective DFT reference E^{ref} and $F^{\mathrm{ref}}_{\alpha,n}$. The color represents the data point density in a grid of size 200×133, which covers the plot area. The training was performed with the CoRe optimizer and lifelong adaptive data selection for 2500 epochs using learning on a stationary batch of all training data.

[1] M. Eckhoff, F. Schönewald, M. Risch, C. A. Volkert, P. E. Blöchl, and J. Behler, Closing the gap between theory and experiment for lithium manganese oxide spinels using a high-dimensional neural network potential, Phys. Rev. B **102**, 174102 (2020).

[2] M. Eckhoff, K. N. Lausch, P. E. Blöchl, and J. Behler, Predicting oxidation and spin states by high-dimensional neural networks: Applications to lithium manganese oxide spinels, J. Chem. Phys. **153**, 164107 (2020).
